# Supplementary material for: Gastric Enteric Glial Cells: A New Contributor to the Synucleinopathies in the MPTP-Induced Parkinsonism Mouse
Source: Molecules. 2022 Nov 1;27(21):7414. doi: 10.3390/molecules27217414 (PMC9656042; doi:10.3390/molecules27217414)
Supplement: Supplementary file 1 [file molecules-27-07414-s001.zip › molecules-1983521-supplementary.pdf]

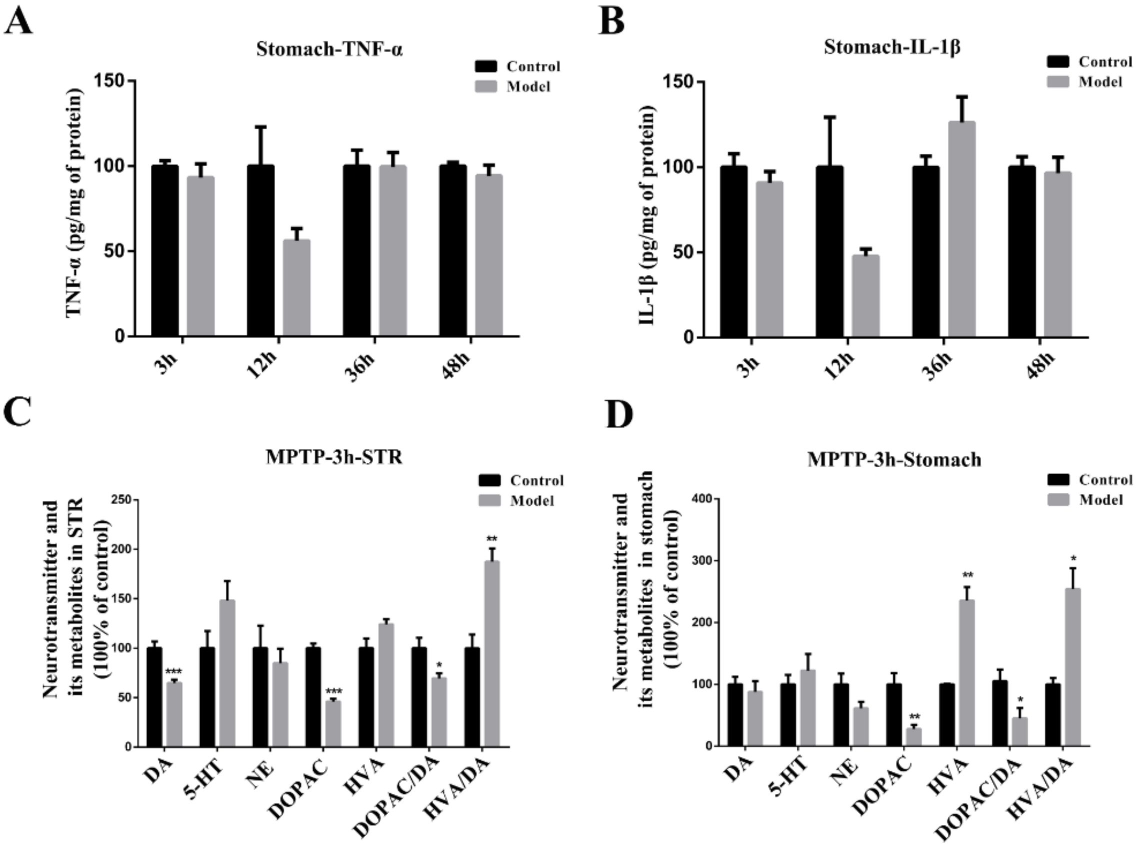

Figure S1. Cytokine and neurotransmitter changes in the stomachs of the single MPTP injection model. Histograms representing the levels of a TNF-α and b IL-1β normalized to total protein levels 3 h, 12 h, 36 h, and 48 h after a single MPTP injection in the stomach measured by ELISA are shown (n = 4). Histograms representing the levels of the main neuro-transmitters and their metabolites 3 h after MPTP injection in the c STR and d stomach across groups are shown (n = 5). The data are presented as the means ± SEM, * $p < 0.05$, ** $p < 0.01$ compared with the control group.
